# Evaluation of porcine GM-CSF during PRRSV infection *in vitro* and *in vivo* indicating a protective role of GM-CSF related with M1 biased activation in alveolar macrophage during PRRSV infection

**DOI:** 10.3389/fimmu.2022.967338

**Published:** 2022-10-19

**Authors:** Qi Ji, Guanggang Qu, Bing Liu, Yang Bai, Guihua Wang, Rui Chen, Xu Zheng, Zhigang Zhang, Yonglin Yang, Chunyan Wu

**Affiliations:** ^1^ Department of Preventive Veterinary Medicine, College of Veterinary Medicine, Northwest Agriculture & Forestry (A&F) University, Yangling, China; ^2^ Shandong Binzhou Animal Science and Veterinary Medicine Academy, Binzhou, China; ^3^ College of Life Science, Northwest Agriculture & Forestry (A&F) University, Yangling, China; ^4^ Weinan Animal Disease Prevention and Control Center, Weinan, China; ^5^ Shaanxi Innolever Biotechnology Co., Ltd., Yangling, China; ^6^ Department of Infectious Diseases, The Affiliated Taizhou People’s Hospital of Nanjing Medical University, Taizhou, China

**Keywords:** PRRSV, GM-CSF, macrophage activation, immune response, ELISA

## Abstract

Granulocyte-macrophage colony stimulating factor (GM-CSF), participates in diverse biological processes associated with innate and adaptive immunity, has unknown effects during PRRSV infection. Here, a double-antibody sandwich ELISA for pGM-CSF was developed in-house for evaluation of pGM-CSF level during PRRSV infection both *in vitro* and *in vivo*. In *in vitro* assay, it was notable that PRRSV-infected porcine alveolar macrophages (PAMs) yielded inconsistent pGM-CSF protein- and mRNA-level, suggesting a post-transcriptional inhibition of pGM-CSF mRNA was employed by PRRSV. Meanwhile, concurrent analysis of pGM-CSF levels in serum samples from PRRSV-infected piglets suggested that effect of PRRSV infection demonstrated minimum effect on pGM-CSF levels regardless of PRRSV virulence phenotypes. Moreover, *in vitro* treatment of PAMs with pGM-CSF prior PRRSV inoculation did not inhibit PRRSV replication in PAMs although genes downstream of pGM-CSF in PAMs could be upregulated by pGM-CSF treatment. Meanwhile, knockdown of pGM-CSF using siRNA did not enhance PRRSV replication as well. Intriguingly, therapeutic antibody treatment of HP-PRRSV-infected piglets led to significantly increased serum pGM-CSF levels, thus aligning with low pneumonia incidence and low intracellular PRRSV-RNA levels in PAMs of therapeutic antibody treated piglets. Furthermore, transcriptome analysis of PAMs from infected piglets revealed increased serum pGM-CSF levels correlated with activation of downstream signal of pGM-CSF in PAMs as evidenced by a M1-like phenotypes of gene expression pattern, implying a potential host-protective role played by pGM-CSF for PRRSV infection *in vivo*. In conclusion, our results demonstrated developments of a highly sensitive and specific ELISA for pGM-CSF and revealed a potential protective role conferred by pGM-CSF during PRRSV infection.

## Introduction

Porcine reproductive and respiratory syndrome virus (PRRSV) is an enveloped positive-strand RNA virus belonging to the genus *Porartevirus* (1, 2), family *Arteriviridae*, and order *Nidovirales* (3). Since discovery, PRRSV has been recognized as one of the most notorious swine pathogens (4, 5). The genome of PRRSV is about 15 kb in size and contains at least 10 open reading frames (ORFs) (3). The two known species of PRRSV, *Betaarterivirus suid* 1 (formally designated *PRRSV-1*) and *Betaarterivirus suid* 2 (formally designated *PRRSV-2*), are serotypically distinct and share only approximately 60% nucleotide sequence identity with each other (6–8). Nevertheless, overall disease phenotype, gross clinical signs, and genomic organization are similar between the two PRRSV species (9).

Accumulating evidence suggests that PRRSVs of both species escape from host innate and adaptive immunity *via* multiple strategies (10, 11), including virally induced microenvironmental cytokine profile modulation as a crucial host-evasive tactic. For instance, inhibition of IFN-α/β or IFN-γ production enhances PRRSV replication, while also ameliorating CTL activation (12–14). Other mechanisms include excessive pro-inflammatory cytokine responses (used by HP-PRRSV) that can induce cytokine storm (15–17) or virus induction of anti-inflammatory cytokine IL-10 production that supports persistent PRRSV infection and viremia (18).

Granulocyte-macrophage colony stimulating factor (GM-CSF) was first identified as an inducer of differentiation and proliferation of granulocytes and macrophages derived from hematopoietic progenitor cells (19). Since then, a variety of immune cells, fibroblasts, and endothelial cells have been shown to produce GM-CSF (20, 21). Indeed, GM-CSF has been shown to participate in a wide range of biological processes associated with innate and adaptive immunity. Although some researches had tried to elucidate the role played by GM-CSF during PRRSV infection, exact role of GM-CSF involved in PRRSV pathogenesis remains unsolved. A previous research demonstrated that piglets inoculated with a recombinant live attenuated PRRSV vaccine (MLV) bearing ORF of GM-CSF (pGM-CSF) exhibited lower viremia, fewer gross lung lesions and higher serum level of IFN-γ as compared to that of piglets receiving MLV alone, whereas humoral response were unchanged (22). Meanwhile, immunization of piglets with adenovirus vectored PRRSV-GP3/GP5 with pGM-CSF induce significantly higher PRRSV-specific neutralizing antibodies and increased the secretion of IFN-γ and IL-4 in piglets’ sera (23). These results indicated that both humoral and cellular immune responses elicited by PRRSV vaccines could be enhanced in the presence of GM-CSF. However, results of another *in vitro* research demonstrated that CD163, the essential receptor for PRRSV infection, could be upregulated in macrophages or monocytes in the presence of pGM-CSF (24). This result was consistent with a more recent study demonstrating that increased expression of CD163 in cultured peripheral blood mononuclear cells (PBMCs) enhanced cell susceptibility to PRRSV-2 (25), implying pGM-CSF may promote infection of macrophages by PRRSV-2 isolates. By contrast, stimulation of *PRRSV-1*-infected monocytes with GM-GSF did not increase cell susceptibility to PRRSV infection (26).

Although little is known regarding pGM-CSF functions in infectious porcine disease, human GM-CSF (hGM-CSF) has been studied extensively and appears to possess both stimulatory and suppressive functions that can influence disease processes associated with autoimmunity, inflammation and cancer (27, 28). Indeed, targeting of GM-CSF has been considered a potential strategy for use in clinical treatment of several autoimmune diseases (29, 30). For example, hGM-CSF-stimulated dendritic cells (DCs) may differentiate into DCs that possess a regulatory phenotype that can promote development of regulatory T cells and production of anti-inflammatory cytokine IL-10 (29, 31). However, effects of pGM-CSF regulatory functions in promoting or impeding PRRSV infection have not yet been investigated.

In this study, a double-antibody sandwich ELISA incorporating with our homemade mouse monoclonal antibody (mAb) and rabbit polyclonal antibody against pGM-CSF was generated and demonstrated higher sensitivity than commercial ELISA kits. It is notable that PRRSV infection of its natural target cells, porcine alveolar macrophages (PAMs), induced a higher level of pGM-CSF mRNA expression as compared to that of non-infected PAMs. Nevertheless, pGM-CSF protein was not detected in either western blots or ELISA, suggesting that a PRRSV infection-induced post-transcriptional control mechanism blocked protein-translation of pGM-CSF mRNA in PAMs, these observations were also consisted with *in vivo* PRRSV infection results. Meanwhile, *in vitro* treatment of PAMs with pGM-CSF prior PRRSV inoculation or knockdown of pGM-CSF using siRNA did not inhibit PRRSV replication although genes downstream of pGM-CSF in PAMs could be upregulated by pGM-CSF treatment. However, application of a PRRSV-specific broad-spectrum neutralizing mAb to treat *in vivo* PRRSV infection of piglets led to significantly increased serum pGM-CSF levels. Thus, pGM-CSF may play a positive role in controlling PRRSV infection and/or in enhancing PRRSV clearance. Moreover, protective role of pGM-CSF in PAMs may be PAMs-dependent, as evidenced by activation of pGM-CSF-dependent genes in PAMs. Taken together, in the present study a pGM-CSF ELISA was developed, applied, and shown to be a suitable tool for evaluating pGM-CSF levels. Importantly, ELISA results revealed that pGM-CSF level was not influenced by PRRSV infection *in vivo*. Nevertheless, a high serum pGM-CSF level was positively correlated with PRRSV clearance and inhibition of viral replication, implying that pGM-CSF may play protective roles during PRRSV infection.

## Materials and methods

### Cells virus and chemicals

MARC-145 and BHK21 cells were maintained in Dulbecco’s Modified Eagle Medium (DMEM, Thermo Fisher Scientific, Waltham, MA, USA) supplemented with 10% fetal bovine serum (FBS) (v/v; Thermo Fisher Scientific), penicillin (100 U/ml), and streptomycin (100 μg/ml). HEK-293T cells were maintained in DMEM as described above for MARC-145 and BHK-21 cell cultures. Porcine alveolar macrophages (PAMs) were prepared from bronchoalveolar lavage of 4-week-old PRRSV-negative pigs as previously described (32). PAMs were maintained in RPMI 1640 medium (Thermo Fisher Scientific) supplemented with 10% FBS (Thermo Fisher Scientific), penicillin (100 U/ml), and streptomycin (100 μg/ml). Bone marrow derived dendritic cells (BM-DCs) were generated using a previous described protocol and maintained under the same condition as PAMs (33).

PRRSV strain VR2385 (GenBank: JX044140.1) was recovered from infectious clone pIR-VR2385-CA as previously described (34, 35). The other PRRSV strains used in this study included two highly pathogenic PRRSV isolates SD16 (GenBank: JX087437.1) and JXA1 (GenBank: EF112445.1), NADC30-like Chinese isolate HNhx (GenBank: KX766379), VR-2332 (GenBank: EF536003.1), and HP-PRRSV-derived modified live virus (MLV) vaccine strain TJM-F92. All PRRSV strains were propagated and titrated in MARC-145 cells or PAMs as previous reported (35).

The siRNAs used for knock-down pGM-CSF expression were artificially synthesized by GenePharma Co., Ltd (Shanghai, China). The sequence of siRNAs used in this study was listed as **Supplementary Table 1**. Transfection of siRNA were conducted using Lipofectamine™ 3000 (Thermo Fisher Scientific) at a siRNA to transfection agent ratio of 1 to 4.

### RNA isolation, reverse transcription, plasmid construction, and real-time quantitative PCR

Porcine peripheral blood mononuclear cells (PBMCs) were isolated from fresh blood (after addition of anticoagulation factor) using Ficoll^®^ Paque Plus (GE healthcare, Chicago, IL, USA). PBMCs were seeded into wells of 12-well plates at a density of 2 × 10^6^ cells/well, stimulated with LPS (*In vivo*gen, San Diego, CA, USA) at a concentration of 0.25 mg/mL, then cultured for 24 h. After cells were harvested, total RNA was isolated using RNAiso Plus (TaKaRa, Dalian, China) then RNA was reverse transcribed using the PrimeScript^®^ RT reagent Kit (TaKaRa) according to the manufacturer’s instructions. The pGM-CSF sequence was amplified from cDNA of PBMCs using Q5^®^ High-Fidelity DNA Polymerase (New England Biolabs, Ipswich, MA, USA) and ligated to pET-28a vector to encode a fusion protein comprised of GM-CSF with the 8×His-tag at its C-terminal. After verification of construct correctness using DNA sequencing, it was transformed into *Escherichia coli* strain BL21 (DE3) for recombinant protein expression. For transient expression in a mammalian system, the cDNA sequence of pGM-CSF was cloned into EcoRI and NotI sites of the VenusC1 vector then expressed as a Venus-fused protein. Transfection of plasmid into HEK-293T cells was conducted using FuGENE^®^ HD Transfection Reagent (Promega, Madison, WI, USA) according to the manufacturer’s instructions.

For qPCR-based evaluation of relative expression of target genes, total RNA was extracted from PAMs using TRizol Reagent (Thermo Fisher Scientific) in accordance with the manufacturer’s instructions. Reverse transcription and qPCR were conducted using a PrimeScript RT reagent Kit (TaKaRa) and 2×RealStar Power SYBR Mixture (Genstar, Beijing, China) as previously described (35). Transcripts of GAPDH were also amplified in parallel for use in normalizing total RNA input. Relative quantification of target genes was calculated using the 2^−ΔΔCt^ method. For quantification of PRRSV-RNA copy numbers in serum samples or PAMs, Taqman probe synthesized by Tsingke Biotech (Beijing, China) was used and qPCR was conducted using PerfectStarII Probe qPCR SuperMix (Transgene, Beijing, China). The pBAC-PRRSV-SD16 infectious clone was used for standard curve calculations. Sequences of primers and the Taqman probe used in this study were listed in **Supplementary Table 2**.

### Expression of recombinant proteins

The pET-28a-pGM-CSF plasmid containing cDNA of pGM-CSF fused to DNA encoding a C-terminal 8×His tag was transformed into cells of *E. coli* strain BL21 (DE3) followed by culturing of cells in LB medium at 37°C for 1.5 h until the OD value of the bacterial culture reached 0.6 to 0.8. Next, protein expression was induced by addition of isopropyl β-D-thiogalactoside (IPTG, 0.5 mM) followed by culturing of bacteria at 37°C for an additional 6 h. After IPTG induction, pelleting of bacteria, and sonication of bacterial cells, inclusion bodies remained that contained recombinant pGM-CSF-8×His. After washing of inclusion bodies with phosphate-buffered saline (PBS), inclusion body proteins were reconstituted in 8 M urea (Sigma-Aldrich, St. Louis, MO, USA) then pGM-CSF-8×His was purified using Ni+ affinity chromatography (Transgene). Dialysis of eluted recombinant pGM-CSF-8×His was conducted stepwise against a series of solutions with decreasing urea concentrations until the dialysis buffer was completely replaced with PBS. Dialyzed proteins in PBS were quantified using a BCA protein assay kit (Thermo Fisher Scientific).

### Ethics statement, immunization, cell fusion, and antibodies production

Six-week-old Balb/C mice and healthy 3-month-old New Zealand White rabbits were obtained from Dashuo Biotech (Chengdu, Sichuan, China). Protocols used for animal handling and experimentation were reviewed and approved by the Animal Welfare Committee of Northwest A&F University (No. CVM-2019-M&R03). All animals were monitored on a daily basis for clinical signs. Every effort was made to minimize suffering of animals such that animals were euthanized to ensure a humane endpoint was reached according to our protocol. Experimental procedures for immunization of mice and fusion of splenocytes with S/p20 cells were conducted as previously described (36). Selected positive hybridoma clones were subjected to subcloning *via* limited dilution and antibody isotyping was conducted using Mouse Monoclonal Antibody Isotyping Reagents (Sigma-Aldrich) according to the manufacturer’s instructions.

Rabbits were immunized with 100 μg of recombinant pGM-CSF-8×His (2 mg/mL) mixed with an equal volume of adjuvant for each immunization, with immunizations repeated a total of 5 times separated by two-week intervals. Freund’s Complete Adjuvant (Sigma-Aldrich) was used for the primary immunization, while Freund’s Incomplete Adjuvant (Sigma-Aldrich) was used for the remaining immunizations. Serum was collected after it met the minimum antiserum titer cutoff of 1:106 as determined *via* enzyme-linked immune immunosorbent assay (ELISA). CNBr-activated Sepharose 4B (GE Healthcare) was washed with a cold solution of 1 mM HCl then was conjugated with recombinant pGM-CSF protein following the manufacturer’s instructions using a ratio of 1 mL (Sepharose 4B) to 10 mg (pGM-CSF protein). Rabbit serum previously filtered through a 0.45-μm membrane (Millipore, Merck, Germany) was added to pGM-CSF-conjugated Sepharose 4B then bound antibodies were eluted using 0.1 M glycine (pH 2.7). Eluted antibody was further dialyzed in PBS buffer overnight at 4°C then antibody concentration was measured at a wavelength of 280 nm.

### Western blot analysis

Recombinant pGM-CSF or BHK21 cells transfected with VenusC1-pGM-CSF were suspended in 1×Laemmli Sample Buffer (Bio-Rad Laboratories, Hercules, CA, USA). Constituent proteins were separated by sodium dodecyl sulfate-polyacrylamide gel electrophoresis (SDS-PAGE) then transferred onto PVDF membranes (Millipore) for WB as previously described (32). Membranes were blocked with 1% BSA in PBS and probed with our home-made antibodies against pGM-CSF (mAb-2A4H11 or rabbit polyclonal Abs) and anti-GFP rabbit polyclonal antibody (Proteintech, Wuhan, China). A home-made PRRSV-N specific mAb-6D10 and commercial tubulin mAb (Transgene) were used for detection of corresponding target. Specific binding of antibodies to corresponding targets was detected using horseradish peroxidase (HRP)-conjugated secondary antibodies (Transgene) and visualized using ECL substrate (Bio-Rad Laboratories). Chemiluminescence signals were recorded digitally using a ChemiDoc™ MP system (Bio-Rad Laboratories). Digital signal acquisition and densitometry analyses were conducted with the ImageLab Program, Version 5.1 (Bio-Rad Laboratories).

### Immunofluorescence assay

BHK21 cells transfected with VenusC1-pGM-CSF or empty vector were fixed with 4% paraformaldehyde (Sigma-Aldrich) and permeabilized with PBS containing 0.5% Triton X-100 (Sigma-Aldrich), then blocked with PBS containing 1% BSA (Sigma-Aldrich). Purified mAb against pGM-CSF (mAb-2A4H11) was used to probe transfected BHK21 cells. Specific interactions between antibody and corresponding target were detected using Alexa Fluor^®^ 555 labeled goat anti-mouse IgG conjugate (Thermo Fisher Scientific). Coverslips were mounted onto slides using ProLong^®^ Gold Antifade Reagent containing 4’,6-diamidino-2-phenylindole (DAPI) (Thermo Fisher Scientific) and observed using a Leica DM1000 fluorescence microscope (Leica Microsystems, Wetzlar, Germany). All images were captured and processed using Leica Application Suite X (Version 1.0, Leica Microsystems).

### Development of a sandwich ELISA for pGM-CSF

The 96-well Polystyrene Microplates (Corning Inc., Corning, NY, USA) were coated with 1 μg/well of mAb-2A4H11 in 100 μL of ELISA coating buffer (carbonate buffer, pH = 9.5-9.6) at 4°C overnight. Unbound mAb was removed by washing wells with PBS-T buffer and wells were further blocked by addition of PBS-T buffer containing 5% skim milk for 3 hours at 37°C. Samples were diluted 20-fold in PBS-T dilution in a final volume of 100 μL before adding to mAb-coated microplates and followed by incubation for 1 hour at 37°C. After washing with PBS-T buffer for three times, 100 μL of PBS-diluted rabbit anti-pGM-CSF polyclonal antibody (1.6 μg/mL) was added to wells followed by incubation for 1 h at 37°C. Interaction between anti-pGM-CSF polyclonal antibody and target were detected by addition of HRP-conjugated goat anti-rabbit secondary antibodies (Transgene) (1:5,000 dilution in PBS) followed by incubation of plates for 1 h at 37°C. Results were visualized after addition of 3,3’,5,5’-tetramethylbenzidine (TMB) substrate (Tiangen Biotech, Beijing, China). A standard curve for the pGM-CSF sandwich ELISA was created using dilutions of recombinant pGM-CSF protein of known concentrations (2000, 1000, 500, 333, 111, 55.5, 27.75, 13.875, 0 pg/mL) that was plotted using GraphPad Prism version 5.0 (GraphPad Software, San Diego, CA, USA). The specificity of the pGM-CSF sandwich ELISA was analyzed using 300 pg of recombinant pGM-CSF protein in a volume of 100 μL. The results were compared to results obtained using equivalent concentrations of recombinant porcine IFN-α (PBL Assay Science, Piscataway, NJ, USA), porcine IFN-β (obtained from a porcine IFN-β ELISA kit, Solarbio Life Science, Beijing China), porcine IFN-γ (R&D system, Minneapolis, MN, USA), and porcine IFN-λ3 (homemade). Absorbances of wells were measured using a VictorX5™ Multilabel Plate Reader (Perkin Elmer, Waltham, MA, USA) at a wavelength of 450 nm.

- Measurement of repeatability of the pGM-CSF sandwich ELISA. The repeatability of the double-antibody sandwich ELISA was assessed by including positive samples as an internal control within each plate. Within-plate precision was calculated from 20 replicates on one plate and inter-plate precision was calculated from a single sample tested using 10 different plates (runs). Repeatability was assessed from coefficient of variation (CV) values (CV = SD/Mean). %CV, means, and standard deviations (SDs) were calculated using a previously reported protocol (37). Generally, a %CV value less than 15 was deemed acceptable.
- Measurement of reactivity. HEK-293T cells were transfected with VenusC1-pGM-CSF or empty vector for 72 h then cells were washed three times with PBS. Next, cells were lysed using PBS containing 0.5% Triton X-100 (Sigma-Aldrich) and 1× protease inhibitor cocktail (Roche, Basel, Switzerland). Cell lysate was clarified by centrifugation at 15,000 ×g for 10 min at 4°C then supernatants were transferred to fresh tubes. Next, supernatants were added to wells of antibody-coated microplates followed by incubation of plates for 1 h at 37°C before homemade anti-pGM-CSF rabbit polyclonal antibody were added. After washing wells using TBS-T buffer for three times, anti-rabbit HRP-conjugate (Transgene) was added followed by visualization using TMB substrate (Tiangen Biotech). Absorbances of each wells were measured using a VictorX5™ Multilabel Plate Reader (Perkin Elmer) using a wavelength of 450 nm.
- Evaluation of test samples. Cell supernatants from cultures of PAMs infected with various PRRSV isolates were collected for further testing to detect and quantify pGM-CSF levels using the pGM-CSF double-antibody sandwich ELISA developed here. Next, to test whether the ELISA could be used to measure amounts of pGM-CSF in *in vivo* samples, serum samples from piglets were diluted for 20-fold in PBS-T buffer before conducting the ELISA.

For evaluation of porcine IFN-γ and IL-13 from serum samples, commercial ELISA kit for IFN-γ (Thermo Fisher Scientific) and IL-13 (Ray Biotech, Norcross, GA, United States) were purchased and conducted following manufacturers’ instruction.

### PRRSV challenge experiments and transcriptome analysis

For PRRSV *in vivo* challenge experiment, four-weeks-old piglets were obtained from a PRRSV-free pig farm near Yangling, Shannxi and further screened for CSFV, PRRSV, PCV2 and ASFV along with corresponding antibodies. Only piglets (n=15) negative for all examined pathogens and antibodies against PRRSV and ASFV were selected. Piglets were randomly divided into three groups (n=5) and housed in separate rooms. One group was inoculated with HP-PRRSV-XJA1 whereas other two groups were inoculated with two attenuated PRRSV vaccines: Ingelvac PRRS MLV (herein named MLV) and TJM-F92 representing vaccines against classic PRRSV and HP-PRRSV, respectively. On the other hand, the animal experiments involving challenge with highly pathogenic PRRSV-JXA1 and administration of a PRRSV-specific broad-spectrum neutralizing mAb as treatment for PRRSV infection of piglets were conducted in our previous report (38). Briefly, lung tissue samples, serum samples, and PAMs were collected from groups of animals challenged with PRRSV-JXA1, animals receiving therapeutic mAb-PN9cx3 after HP-PRRSV-JXA1 inoculation, animals receiving control mAb after HP-PRRSV-JXA1 inoculation, and healthy control group (MOCK). Lung tissues collected from piglets at 21 dpi were subjected to histopathologic examination using standard protocols. Briefly, all tissue samples were fixed with 10% neutral buffered formalin then were embedded in paraffin and sectioned for use in histological procedures. Sections stained with hematoxylin and eosin (H&E) were microscopically examined to detect micropathological changes.

Fresh PAMs harvested from piglets were centrifuged at 300 ×g for 10 min. Next, 3 × 10^7^ cells were lysed in 3 mL of TRizol Reagent (Thermo Fisher Scientific) for RNA purification, qPCR, and transcriptome analysis. RNA-seq was conducted by GENEWIZ Co., Ltd. (Suzhou, China). RNA-seq libraries were generated using the Illumina system (RS-122-2201) according to the manufacturer’s protocols. The reference pig genome assembly (*Sus scrofa* v11.1) was downloaded from the Ensemble website. Transcript abundances were converted to transcripts per million (TPM) units using Kallisto then the TPM value for a given gene was defined using the most abundant transcript associated with that particular gene. Transcript abundances of selected genes were used to generate heatmaps, which were drawn using the R package tool “pheatmap” from The R Project for Statistical Computing. RNA-seq datasets generated in this study can be found in the aforementioned NCBI Gene Expression Omnibus (GEO Accession No. GSE156504).

### Statistical analysis

Statistical analysis was performed using GraphPad Prism version 5.0 (GraphPad Software). Indicator differences between treatment groups and controls were assessed using Student’s t-test. A two-tailed P-value of <0.05 was considered statistically significant and a *P*-value of <0.01 was considered extremely significant.

## Results

### Development of pGM-CSF double-antibody sandwich ELISA

**Figure 1:**
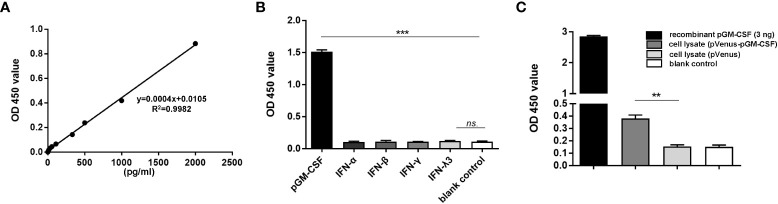
Evaluation of specificity and repeatability for pGM-CSF double antibody sandwich ELISA. **(A)** The standard curve was generated by using recombinant pGM-CSF protein at the indicated concentrations, the formula and correlation coefficient were shown as well. **(B)** Evaluation of the assay specificity of pGM-CSF ELISA. Recombinant pGM-CSF along with IFN-α, IFN-β, IFN-γ, and IFN-λ3 at the concentration of 3ng/ml was used for pGM-CSF double antibody sandwich ELISA to evaluate the specificity the assay. **(C)** Evaluation of the pGM-CSF ELISA reactivity with mammalian expressed pGM-CSF. HEK-293T cells transfected with VenusC1-pGM-CSF or empty vector for 24 hours, then cells were lyzed using NP40 buffer and added as ELISA sample for pGM-CSF detection, and recombinant pGM-CSF protein (3ng) was included as positive control. Experiments were repeated at least three times. All data are expressed as mean ± SD and were subjected to Student’s t-test. Significant differences between indicated groups are marked by “***”, which means *p*< 0.001; ** means *p*< 0.01.

To obtain antibodies for development of pGM-CSF ELISA with higher sensitivity, cDNA sequence of pGM-CSF was cloned from LPS-stimulated PBMCs (**Figure S1A**) and ligated to pET-28a vector for recombinant expression (**Figure S1B**). After the immunization and cell fusion, followed by screening of surviving hybridomas, a hybridoma clone (mAb-2A4H11, IgG1), was generated and further verified for its reactivity with mammalian cells transfected with pGM-CSF fused with Venus protein in IFA (**Figure S2A**) and WB (**Figure S2B**). Moreover, purified rabbit polyclonal antibody against recombinant pGM-CSF were generated and tested for reactivity with pGM-CSF (**Figure S2B**). Next, by employing mAb-2A4H11 and rabbit polyclonal antibody as coating antibody and detection antibody, a double-antibody sandwich ELISA to detect pGM-CSF was developed. Based on the generated standard curve. Regression analysis of the data yielded an acceptable correlation coefficient (R2 = 0.9982) and a minimum detection limit of 27.75 pg/mL (**Figure 1A**).

To evaluate the specificity of the pGM-CSF ELISA, we tested its reactivity to other recombinant cytokines with porcine origin (IFN-α, β, γ, λ). As demonstrated in **Figure 1B**, the sandwich ELISA cannot detect recombinant porcine IFN-α, β, γ, or λ, but only detect pGM-CSF, as reflected by significantly increased OD values associated with wells containing recombinant pGM-CSF (*p <* 0.001, **Figure 1B**), suggesting this ELISA method is highly specific to pGM-CSF. Next, the pGM-CSF double-antibody sandwich ELISA was tested to determine its repeatability. Repeatability is important for assessing suitability of the pGM-CSF sandwich ELISA for use in diagnostic applications for quantifying GM-CSF in serum samples with known pGM-CSF concentrations. The ELISA results demonstrated a within plate %CV of 5.33 and an inter-plate %CV (between different runs) of 5.41 (**Table 1**), thus demonstrating that the pGM-CSF double-antibody sandwich ELISA was highly repeatable. Taken together, these testing data demonstrated high specificity, sensitivity, and repeatability for our homemade pGM-CSF sandwich ELISA. Furthermore, a lysate of HEK-293T cells transfected with VenusC1-pGM-CSF or empty vector were tested *via* the ELISA to confirm that mAb-2A4H11 could capture pGM-CSF expressed in mammalian cells as well. As compared with lysates of cells transfected with empty vector or blank control, the pGM-CSF ELISA detected significant pGM-CSF expression based on OD values obtained for lysates of VenusC1-pGM-CSF-transfected mammalian cells (*p* < 0.01, **Figure 1C**).

**Table 1:** Assay repeatability.

Assay	Repeatability result (% CV)	
	Within plate	Between runs
pGM-CSF Double Antibody Sandwich ELISA^a^	5.33	5.41

a Bold letter represents a positive serum sample used for testing the repeatability of this assay.

### PRRSV infection of PAMs induced expression of GM-CSF mRNA but not GM-CSF protein

**Figure 2:**
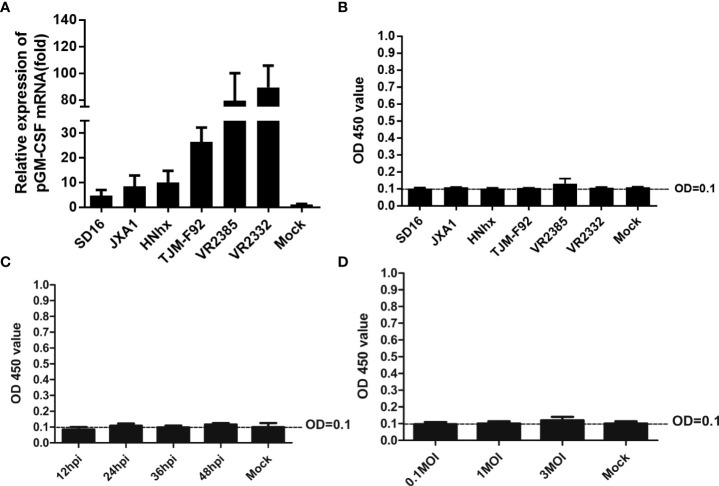
Evaluation of pGM-CSF mRNA and protein expression in PAMs infected by different PRRSV isolates. **(A)** PAMs were infected with infected with heterogeneous PRRSV isolates (PRRSV-JXA1, SD16, NADC30-like strain HNhx, vaccine strain TJM-F92 and classic strains VR2385 and VR2332) at 1 MOI for 24 hours. Then the cells were harvested for qPCR to evaluate the mRNA level of pGM-CSF. Non-infected PAMs served as controls (MOCK). All experiment was repeated at least for three times. **(B)** Cell culture supernatant collected from PAMs infected with heterogeneous PRRSV isolates (PRRSV-JXA1, SD16, NADC30-like strain HNhx, vaccine strain TJM-F92 and classic strains VR2385 and VR2332) at 1 MOI for 24 hours were harvested for ELISA analysis for pGM-CSF proteins. Supernatant from non-infected PAMs served as controls (MOCK). **(C)** Cell culture supernatant collected from PAMs infected with PRRSV-JXA1 at 1 MOI for different time hours were harvested for ELISA analysis for pGM-CSF proteins. Supernatant from non-infected PAMs served as controls (MOCK). **(D)** Cell culture supernatant collected from PAMs infected with PRRSV-JXA1 at different MOIs were harvested for ELISA analysis for pGM-CSF proteins. Supernatant from non-infected PAMs served as controls (MOCK). All experiment was repeated at least for three times and all data are expressed as mean ± SD.

Previous studies had demonstrated that inoculation of piglets with recombinant live attenuated PRRSV expressing pGM-CSF or PRRSV-GP3/GP5 proteins fused with pGM-CSF as recombinant vaccine conferred better protection against virulent PRRSV challenge *in vivo* (22, 23). However, pGM-CSF has also been shown to possess an immune regulatory function whereby its production was strongly linked to amelioration of autoimmune and inflammatory disorders (30). Therefore, it would be interesting to know if pGM-CSF expression can influence the course of PRRSV infection both *in vitro* and *in vivo*. To answer this question, *in vitro* cultured PAMs were infected with heterogeneous PRRSV isolates, including HP-PRRSV strains (SD16 and JXA1), a NADC30-like China isolate HNhx strain, an attenuated HP-PRRSV vaccine strain TJM-F92, and classical strains VR2385 and VR2332. Based on our data, all PRRSV strains induced significant upregulation of pGM-CSF mRNA in PAMs, although several-fold differences were observed among different virus strains (**Figure 2A**). More specifically, these results indicated that lower-level of pGM-CSF mRNA upregulation was observed in PAMs infected with HP-PRRSV and NADC30-like strains as compared to that of PAMs infected by classical PRRSV strains VR2385 and VR2332 (**Figure 2A**). Nevertheless, ELISA results reflecting pGM-CSF protein levels in supernatants collected from PRRSV-infected PAMs unexpectedly detected no pGM-CSF protein (**Figure 2B**). To rule out the possibility that pGM-CSF protein was produced but not released from PRRSV infected cells, PAMs were lysed and cell lysates were evaluated *via* ELISA, with no pGM-CSF detected in PAM lysates (data not shown) as well. Furthermore, the supernatant of PRRSV-JXA1 infected PAMs with different inoculation time (**Figure 2C**) and dose (**Figure 2D**) were subjected to ELISA screening. However, no production of pGM-CSF in supernatants was detected. Therefore, these data demonstrated that PRRSV infection did not induce secretion of pGM-CSF *in vitro* regardless of virulence phenotype of PRRSV used to inoculate PAMs. However, increasing expression of pGM-CSF mRNA could be observed in *in vitro* infected PAMs but no detectable pGM-CSF protein was identified.

### pGM-CSF does not directly inhibit PRRSV infection in PAMs *in vitro*

**Figure 3:**
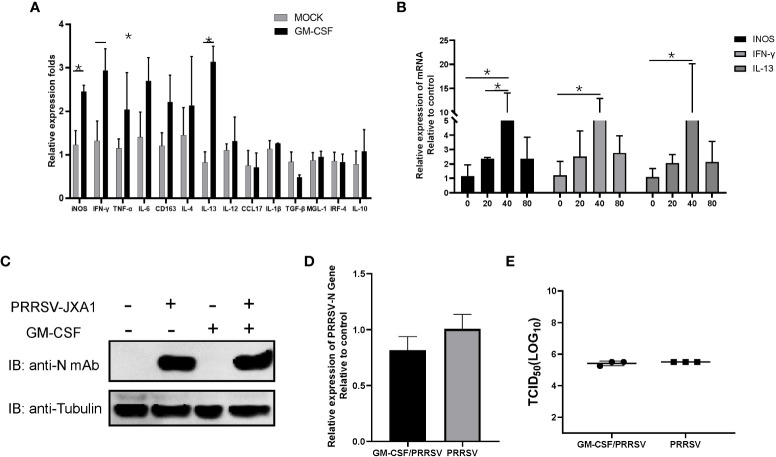
The pGM-CSF cannot inhibit PRRSV replication in PAMs *in vitro*. **(A)** PAMs were either treated with 40ng recombinant pGM-CSF for 24 hours or left untreated (MOCK). Next, PAMs were harvested by TRIzol agent and reverse transcribed. The mRNA level of indicated genes was evaluated using qPCR. Experiments were repeated for at least three times. All data are expressed as mean ± SD and were subjected to Student’s t-test. Significant differences between indicated groups are marked by “*” (*p* < 0.05). **(B)** PAMs were treated with different dose of recombinant pGM-CSF for 24 hours or left untreated (0ng). Next, PAMs were harvested by TRIzol agent and reverse transcribed. The mRNA level of iNOS, IFN-γ and IL-13 was evaluated using qPCR. Experiments were repeated for at least three times. All data are expressed as mean ± SD and subjected to Student’s *t*-test. Significant differences between indicated groups are marked by “*” (*p* < 0.05). **(C)** PAMs were either treated with 40ng recombinant pGM-CSF protein for 24 hours or left un-treated (MOCK) before inoculated with PRRSV-JXA1 strains (0.1 MOI) for additional 24 hours. PAMs were harvested for SDS-PAGE followed by western blotting assay use anti-PRRSV-N specific mAb-6D10. Tubulin was probed from the same membrane as a loading control. **(D)** PAMs were either treated with 40ng recombinant pGM-CSF protein for 24 hours or left un-treated (MOCK) before inoculated with PRRSV-JXA1 strains (0.1 MOI) for additional 24 hours. Next, PAMs were harvested by TRIzol agent and reverse transcribed for evaluating PRRSV-RNA level using qPCR. Experiments were repeated for at least three times and presented as mean ± SD. **(E)** PAMs were either treated with 40ng recombinant pGM-CSF protein for 24 hours or left un-treated (MOCK) before inoculated with PRRSV-JXA1 strains (0.1 MOI) for additional 24 hours. Next, cell culture supernatant of PAMs were harvested for PRRSV titration and determined as TCID_50_. Experiments were repeated for at least three times and presented as mean ± SD.

Alveolar macrophages have been proposed to be major *in vivo* effector cells triggered by GM-CSF during influenza A virus infection but also the major *in vivo* target of PRRSV infection (38, 39). Although *in vitro* infection of PAMs by PRRSV induced inconsistence between pGM-CSF mRNA and protein level, it is interesting to investigate if recombinant pGM-CSF inhibit PRRSV infection *in vitro*. Therefore, 40 ng recombinant pGM-CSF (the commonly used dose for *in vitro* differentiation of bone marrow cells to dendritic cells) was used to treat freshly isolated PAMs cells, qPCR analysis was conducted to understand the transcriptional change of M1-like cytokines (TNF-α, IL-1β, IL-6, IL-12, IFN-γ, iNOS, TGF-β, IRF-4, CCL17) and M2-like cytokines (IL-4, 1L-10, CD163, IL-13, MGL-1), while these genes are typically upregulated in GM-CSF-treated monocytes/macrophages based on previous reports (40, 41). Based on our data, expression of iNOS and IFN-γ (M1 category), and IL-13 (M2 category), demonstrated significantly upregulation in pGM-CSF treated PAMs (*p*<0.05) whereas no significant alternation of mRNA level was observed for other genes in both categories (**Figure 3A**). Meanwhile, the effect of pGM-CSF doses on mRNA expression of iNOS, IFN-γ and IL-13 were further evaluated and our data suggested that 40 ng pGM-CSF treatment inducted highest expression of these genes (**Figure 3B**). Therefore, these data suggested that *in vitro* cultured PAMs deed response for exogenous pGM-CSF stimulation. Next, we examined PRRSV replication in pGM-CSF-treated PAMs. After 24-hours treatment, HP-PRRSV-JXA1 was used to inoculate PAMs. However, exogenous pGM-CSF stimulation neither inhibited nor promoted PRRSV replication in PAMs as determined by evaluation of PRRSV-N protein level (**Figure 3C**). Conversely, the intracellular PRRSV-RNA (**Figure 3D**) and infectious viral particles (**Figure 3E**) of cell culture supernatant were evaluated as well. Based on our data, treatment of PAMs with pGM-CSF neither changed intracellular PRRSV-RNA or infectious viral particles as well (**Figures 3D, E**).

**Figure 4:**
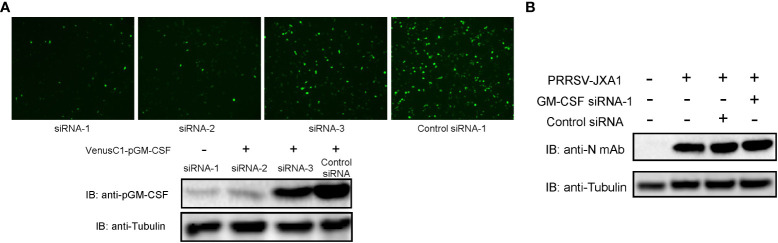
Knock-down of pGM-CSF in BM-DCs did not promote PRRSV replication *in vitro*. **(A)** HEK-293T cells were transfected with pVenusC1-pGM-CSF plasmids for 5 hours, then followed by transfection of 1μg pGM-CSF siRNAs or control siRNA for additional 24 hours. Next, the cells were observed directly under microscope of harvested for western blot using pGM-CSF specific mAb-2A4H11. Tubulin was probed from the same samples and included as protein loading control. **(B)** BM-DCs were either transfected with 1μg pGM-CSF siRNA-1 or control siRNA for 24 hours or left as un-transfected cells before inoculated with PRRSV-JXA1 strains (0.1 MOI) for additional 24 hours. Next, cells were harvested for SDS-PAGE followed by western blotting assay using anti-PRRSV-N specific mAb-6D10. Tubulin was probed from the same membrane as a loading control.

On the contrary, to further confirm that pGM-CSF does not directly inhibit PRRSV replication *in vitro*, siRNA based knock-down assay was conducted in BM-DCs since our previous observation that BM-DCs originated from *in vitro* differentiation of pGM-CSF stimulated Bone marrow cells maintain is susceptibility to PRRSV infection (33). First, 3 different pGM-CSF specific siRNAs were artificially synthesized and tested in VenusC1-pGM-CSF transfected HEK-293T cells for their capability to knock-down pGM-CSF expression since endogenous pGM-CSF cannot be detected in either PAMs and BM-DCs. Based on the result, among 3 siRNAs, siRNA-1 and siRNA-2 transfected cells demonstrated significant reduction of GFP signal (**Figure 4A**), whereas siRNA-3 demonstrated minimum knock-down effect as compared to control siRNA. Meanwhile, western blot analysis further confirmed that siRNA-1 and siRNA-2 can effectively knock-down expression of pGM-CSF expression. Therefore, pGM-CSF specific siRNA-1 was introduced to BM-DCs to knock-down pGM-CSF before PRRSV inoculation, however, it appeared that knock-down of pGM-CSF conferred no effect on PRRSV replication as PRRSV-N protein level was similar among normal cells and BM-DCs transfected with either control siRNA or pGM-CSF-specific siRNA-1 (**Figure 4B**). Taken together, these data suggested that pGM-CSF treatment cannot confer an antiviral state in PAMs to inhibit PRRSV replication and knock-down of pGM-CSF in BM-DCs also cannot promote PRRSV replication, suggesting that PRRSV-susceptible cells could response to exogenous pGM-CSF stimulation but pGM-CSF did not inhibit PRRSV replication directly in PRRSV-susceptible cells.

### PRRSV infection *in vivo* did not lead to pGM-CSF expression regardless of disease phenotype

**Figure 5:**
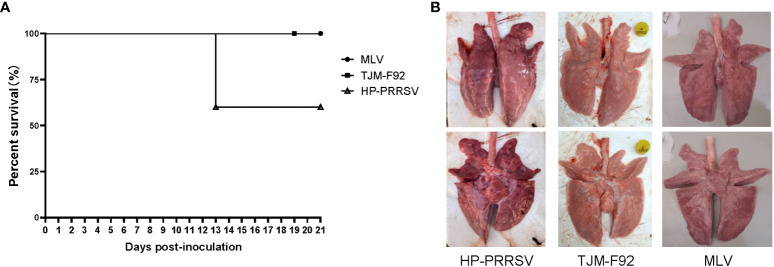
Infection of piglets using PRRSV strains with different virulence cannot induce pGM-CSF induction. **(A)** All piglets were inoculated with 1.0×10^5^ TCID50 of HP-PRRSV-XJA1, or two vaccines strains (MLV and TJM-F92) *via* both intramuscular and intranasal routes. Clinical signs and survival rates were monitored daily for a total of 21 days. **(B)** Representative ventral and dorsal lung images from surviving piglets of each group were captured immediately after piglets were autopsied at 21 dpi.

Although above *in vitro* result suggested an inconsistence between pGM-CSF mRNA and protein level in PRRSV infected PAMs, it is necessary to figure out if PRRSV infection induced pGM-CSF *in vivo* whereas previous reports strongly suggested that pGM-CSF demonstrated dual roles in virus-induced pneumonia as observed in influenza A virus infection and COVID-19 patients (39, 42, 43). Therefore, analysis of serum samples from both virulence PRRSV (HP-PRRSV) and attenuated PRRSVs (MLV and TJM-F92) infected piglets was conducted using homemade pGM-CSF ELISA. As demonstrated in **Figure 5A**, after virus inoculation, beginning at 13 dpi, mortality due to HP-PRRSV challenge emerged with two piglets dead but 3 piglets survived until end of experiment. Meanwhile, 100% surviving rate could be observed in of either MLV or TJM-F92 immunized groups (**Figure 5A**). Meanwhile, lunge sample from HP-PRRSV groups demonstrated extensive pneumonia and severe pathological changes associated with virulent HP-PRRSV challenge (**Figure 5B**). By contrast, lungs of PRRSV-MLV or TJM-F92 vaccinated piglets resembled lungs with minimum pathological changes (**Figure 5B**). However, no matter the disease phenotypes and infecting times (serum samples harvested weekly), pGM-CSF cannot be detected in any serum samples from all groups of piglets (**Table 2**). Taken together, it appeared that PRRSV infection alone *in vivo* cannot lead to the production of pGM-CSF regardless of disease phenotype and virulence phenotype of challenging virus, which appears to be different from the scenario of influenza virus infection or SARS-CoV2 infection (39, 42, 43).

**Table 2:** Evaluation of pGM-CSF level of serum samples inoculated witgh different PRRSV by Double Antibody Sandwich ELISA.

Groups	No. of seropositive animals^a^ detected/total no. of experimental animals		
	7d^b^	14d	21d
HP-PRRSV-XJA1 inoculation	0/5	0/5	0/5
Ingelvac PRRS MLV inoculation	0/5	0/5	0/5
TJM-F92 inoculation	0/5	0/5	0/5

a An S/N value of 2.1 is considered seropositive for pGM-CSF Double Antibody Sandwich ELISA.

b Days post-infection.

### pGM-CSF plays a host-protective role during PRRSV infection of piglets

**Figure 6:**
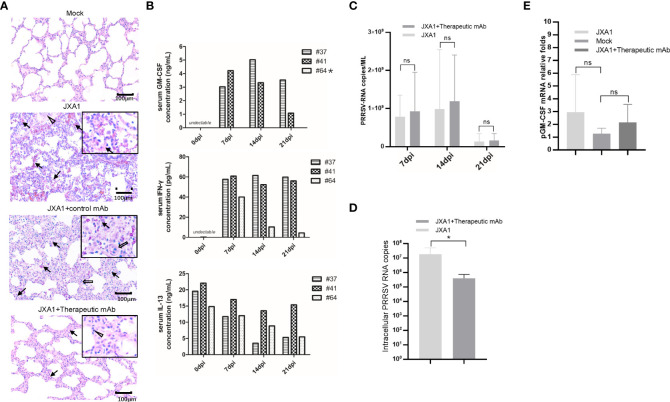
Serum pGM-CSF level in PRRSV infected piglets correlated with disease. **(A)** Lung gross pathological examination *via* hematoxylin and eosin (H&E) staining was conducted for piglets infected with PRRSV-JXA1(JXA1), PRRSV-infected but therapeutic mAb treated piglets (JXA1+therapeutic mAb), or PRRSV-infected but control mAb treated piglets (JXA1+control mAb). Lung tissue sample from non-infected piglets (Mock) was includes as control. Arrow indicated thickening of interlobular septal or infiltration of inflammatory cells around bronchiole or within or around alveolus and bronchus. Triangle indicates inflammatory cells, necrotic debris and exfoliated epithelial cells infiltrate in the bronchiole. Hollow arrow indicated hemorrhage or infiltration of inflammatory cells within alveolar septa, and alveolar spaces. **(B)** Evaluation of serum pGM-CSF, IFN-γ and IL-13 concentration from the piglets inoculated with PRRSV-JXA1 but treated with therapeutic mAb at different time points (7 dpi, 14dpi and 21dpi) using home-made pGM-CSF ELISA method and commercial ELISA kits for IFN-γ and IL-13. Animal number (64) demonstrated no detectable pGM-CSF was marked by “*”. **(C)** Serum samples from piglets were harvested by TRIzol agent for RNA extraction and reverse transcription. The serum PRRSV-RNA copied numbers was evaluated using Taqman probe in qPCR. **(D)** PAMs from piglets were harvested by TRIzol agent for RNA extraction and reverse transcription. The intracellular PRRSV-RNA copied numbers was evaluated using Taqman probe in qPCR. **(E)** PAMs from piglets were harvested by TRIzol for RNA extraction and reverse transcription. The mRNA level of pGM-CSF was evaluated using qPCR. Experiments for above qPCR were repeated at least three times (representing three different animals). All data are presented as mean ± SD and were subjected to Student’s t-test. Significant differences between indicated groups are marked by “*” (*p* < 0.05), whereas “ns” means no sense.

Besides *in vitro* and *in vivo* PRRSV infection, to further investigate *in vivo* effects of pGM-CSF on PRRSV infection processes, we retested serum and PAMs samples obtained from our previous animal study investigating therapeutic potential of a broad-spectrum neutralizing monoclonal antibody against *in vivo* infection cause by heterogeneous PRRSV strains (38). Notably, *in vivo* administration of the therapeutic mAb (mAb-PN9cx3) led to significant reduction of PRRSV infection-induced pathological lung changes (**Figure 6A**), while control mAb had minimal therapeutic effect. Concurrently, use of the ELISA developed here to evaluate pGM-CSF levels in piglet serum samples revealed no detectable pGM-CSF in serum samples collected from piglets of the MOCK (uninfected) group, PRRSV-JXA1-inoculated group, and control mAb-treated group regardless of time point of serum collection (**Table 3**). However, in the therapeutic mAb-treated group, 2 of 3 serum samples were positive for pGM-CSF from 7 dpi to 21 dpi (**Table 3**), suggesting that aside from blocking of PRRSV replication, administration of a therapeutic mAb to piglets may have also contributed to immune system activation that led to increased serum pGM-CSF levels. Details regarding serum levels of pGM-CSF associated with the two GM-CSF-positive animals (#37 and #41) throughout the entire *in vivo* experiment are shown in **Figure 6B**. Besides serum pGM-CSF levels, the serum IFN-γ and IL-13 were evaluated as well using commercial ELISA kits (**Figure 6B**). Similar with the pGM-CSF level, serum IFN-γ could be detected in all three piglets in the therapeutic mAb-treated group, whereas no detectable IFN-γ in serum samples collected from piglets of other groups (**Table 3**). A significant increasing of serum IFN-γ was observed after PRRSV inoculation in therapeutic mAb-treated group (**Figure 6B**). Notably, two GM-CSF-positive piglets (#37 and #41) also demonstrated higher serum IFN-γ level than piglets #64 (GM-CSF negative), suggested that serum pGM-CSF and IFN-γ level may be correlated with each other since IFN-γ is a typical M1/Th1 cytokines. Conversely, serum IL-13 (M2 category) was also investigated as well. While IL-13 could be detected in all piglets’ serum regardless groups (**Table 3**), we further analyzed the kinetics IL-13 in therapeutic mAb-treated group, but no significant changes were observed (**Figure 6B**).

**Table 3:** Detection of cytokine from serum samples.

Groups	No. of seropositive animals^a^ detected/total no. of experimental animals								
	7d^b^			14d			21d		
	pGM-CSF	IFN-γ^C^	IL-13^C^	pGM-CSF	IFN-γ^C^	IL-13^C^	pGM-CSF	IFN-γ^C^	IL-13^C^
JXA1 inoculation	0/3	0/3	3/3	0/3	0/3	3/3	0/3	0/3	3/3
JXA1+ therapeutic mAb	2/3	3/3	3/3	2/3	3/3	3/3	2/3	3/3	3/3
JXA1+control mAb	0/3	0/3	3/3	0/3	0/3	3/3	0/3	0/3	3/3
Mock	0/3	0/3	3/3	0/3	0/3	3/3	0/3	0/3	3/3

a An S/N value of 2.1 is considered seropositive for pGM-CSF Double Antibody Sandwich ELISA.

b Days post-infection.

^C^Evaluated using commercial ELISA kits.

Besides serum cytokines, viral RNA copy numbers in serum samples were also evaluated using a Taqman probe designed against the PRRSV-N protein-encoding sequence. As consistent with our previous observations (38), serum viral RNA levels were similar among groups regardless of serum pGM-CSF level (**Figure 6C**). However, intracellular PRRSV-RNA levels in PAMs from the therapeutic mAb-treated group were significantly lower than corresponding levels in PAMs of the PRRSV-JXA1-inoculated group (**Figure 6D**). This result prompted us to investigate pGM-CSF mRNA levels in PAMs isolated from piglets, with the results revealing no significant change in pGM-CSF mRNA level in PAMs after PRRSV infection. Nevertheless, these is no statistically significant upregulation of pGM-CSF mRNA expression in PAMs of either PRRSV-JXA1-inoculated piglets or therapeutic mAb-treated piglets as compared to PAMs of the MOCK group (**Figure 6E**). Therefore, it is possible that PAMs were not the major *in vivo* source of serum pGM-CSF protein detected in therapeutic mAb-treated piglets.

**Figure 7:**
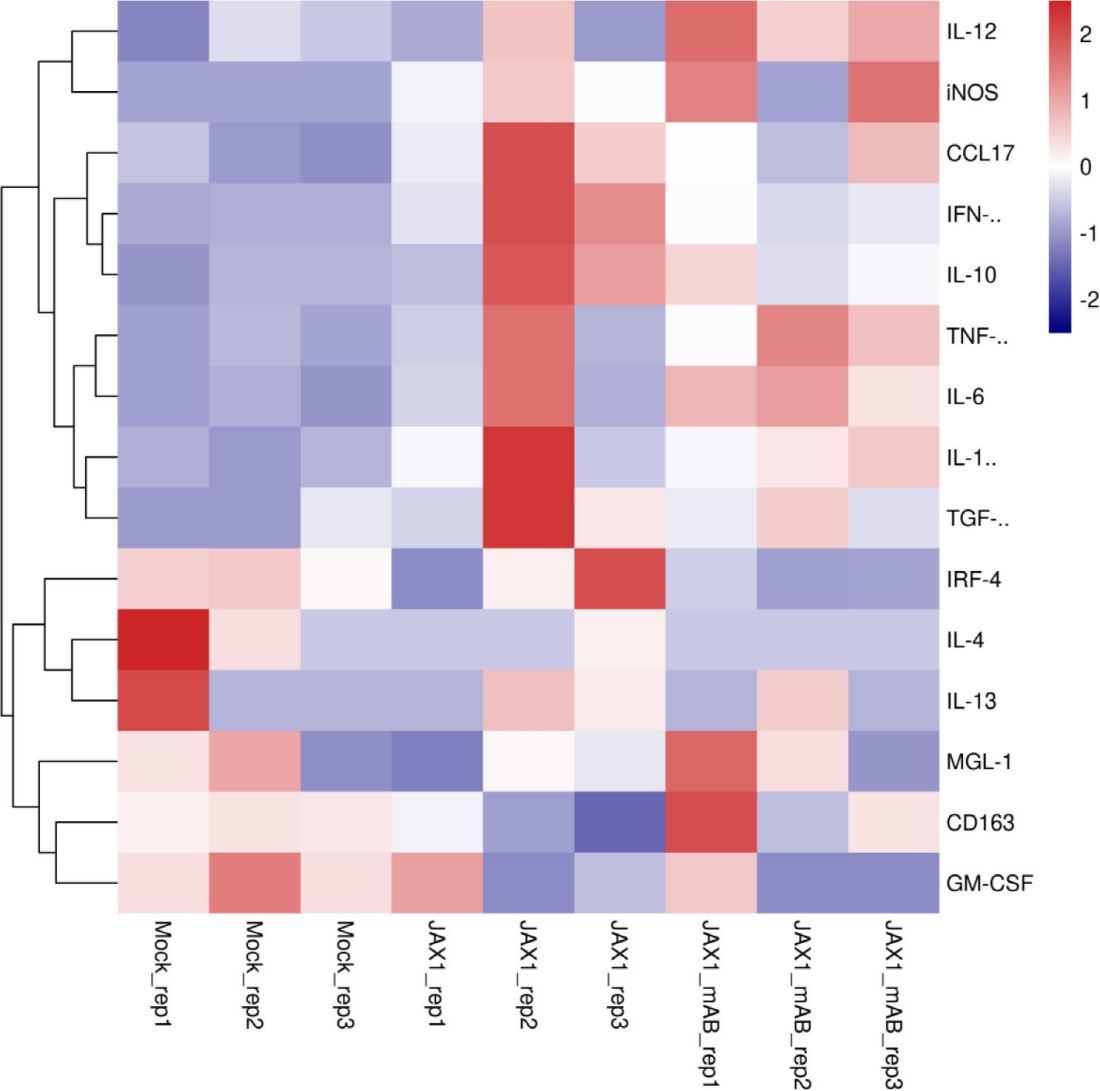
Transcriptome profiling of pGM-CSF activated genes in PAMs isolated from PRRSV-JXA1-infected piglets with or without therapeutic mAb treatment. Heatmap analysis of genes downstream of pGM-CSF from PAMs collected from PRRSV-JXA1 infected piglets (JXA1) and PAMs from PRRSV-JXA1 infected but treated with therapeutic mAb (JXA1_mAb). The health piglets inoculated with PBS was included as control (Mock).

Although pGM-CSF cannot confer antiviral states and inhibit PRRSV replication in PAMs *in vitro* but *in vivo* data supported a potentially protective role played by pGM-CSF, therefore, putative downstream signaling genes (M1-like cytokines and M2-like cytokines) of pGM-CSF along with pGM-CSF itself were selected for transcriptome analysis for PAMs cells isolated from piglets (**Figure 6**). Based on transcriptome analysis, expression of pGM-CSF from PAMs appears to demonstrate a trend of down-regulation with no significant alternation among groups (**Figure 6**), further suggested that PAMs might be not be the *in vivo* source of serum pGM-CSF in therapeutic mAb-treated piglets. However, most M1-like cytokines, such as IL-12, iNOS, TNF-α, IL-1β and IL-6, were upregulated in PAMs collected from therapeutic mAb-treated piglets and consisted with evaluated IFN-γ level as well (**Figure 7**). These results suggested that PAMs in therapeutic mAb-treated piglets might be activated by pGM-CSF, aligning with observed reductions of both pneumonia incidence and intracellular PRRSV-RNA levels. Taken together, these data demonstrated that pGM-CSF might exert a protective role during *in vivo* PRRSV infection by inducing alveolar macrophages to develop M1-like characteristics and provide additional evidence implied that pGM-CSF may hold protective role against PRRSV infection *in vivo*.

## Discussion

Since PRRSV was discovered, substantial efforts have been made to control this pathogen. Nevertheless, PRRSV continues to threaten the global swine industry, due to continuously outbreaks that have outpaced gains in our understanding of PRRSV pathogenesis (44, 45). In humans, both immune stimulatory and suppressive functions have been reported for GM-CSF (27, 28), whereby GM-CSF has served as a promising therapeutic target for clinical treatment of both autoimmune and infectious diseases (29, 30). Meanwhile, other studies proposed that GM-CSF may be useful as a novel vaccine adjuvant to combat human and animal virus, such as rabies virus (46), hantavirus (47, 48), and PRRSV (22, 23), while GM-CSF also acts for potential cancer vaccine (49). Nonetheless, exact roles played by GM-CSF during viral infections, especially respiratory virus infections, remain controversial. On the one hand, overexpression of GM-CSF in lungs of transgenic mice provided remarkable protection against influenza A virus infection-induced pneumonia and appeared to depend on alveolar macrophage function (39). On the other hand, increased percentages of GM-CSF-expressing leukocytes have been found in blood of COVID-19 patients and no clinical benefits of recombinant hGM-CSF treatment have yet been reported for treating COVID-19 patients (43), whereas mAb-induced neutralization of hGM-CSF protein has attracted great interest as an anti-inflammatory therapy for COVID-19 patients (43). Therefore, it appears that GM-CSF plays dual-roles in lung infection-associated pneumonia, or GM-CSF effects on alveolar macrophage homeostasis and lung pathogen clearance may by virus-specific (42).

For PRRSV infection, although porcine alveolar macrophages, the potential effector cells of pGM-CSF, are recognized as the primary target of PRRSV infection *in vivo* (12, 50), the exact role of pGM-CSF in influencing PRRSV infection processes remain unclear due to contradictory data obtained (25, 26). On the one hand, BM-DCs obtained *via in vitro* differentiation from recombinant pGM-CSF-treated bone-marrow cells remains susceptible for PRRSV infection with no correlation was observed between pGM-CSF dose used for BM-DCs differentiation and BM-DCs’ susceptibility for PRRSV (33). On the other hand, previous *in vivo* studies comparing highly pathogenic, classical, and attenuated vaccine PRRSV strains demonstrated that rapid HP-PRRSV replication in pigs could trigger cytokine storm (15–17), and lead to robust inflammatory responses with high mortality, are associated with sustained expression of pro-inflammatory cytokines and chemokines that may include pGM-CSF. However, due to the lack of a highly sensitive ELISA for detecting pGM-CSF, *in vivo* pGM-CSF levels and its kinetics during PRRSV infection of piglets could not be determined.

In this study, a pGM-CSF ELISA was developed for evaluating pGM-CSF levels during PRRSV infection *in vitro* and *in vivo*. Notably, *in vitro* infection of PAMs with diverse PRRSV isolates (classical strain, highly pathogenic strain, vaccine strain) induced significant upregulation of pGM-CSF mRNA levels (**Figure 2A**). However, no pGM-CSF protein could be detected in PAMs using this ELISA assay (**Figure 2B**), suggesting that upregulation of pGM-CSF mRNA in PAMs did not reflect corresponding protein expression trends. Meanwhile, infection of piglets with both virulent and attenuated PRRSV strains did not produce pGM-CSF *in vivo*, which appears to be consisted with *in vitro* result. Researchers observed very early that a discrepancy existed between IFN-α mRNA-level and protein-level expression in PRRSV-infected PAMs (51). This result was further supported by more recent observations that PRRSV infection of monocyte-derived dendritic cells activated transcription of IFN-α/β but failed to generate bio-active IFNs proteins (52). Thus, the scenario of pGM-CSF expression in PRRSV-infected PAMs appears to mirror that observed for IFNs as well (53), implying that a post-transcriptional control mechanism might be employed by PRRSV to block production of IFNs and/or other cytokines (e.g., GM-CSF) in infected PAMs (53). Conversely, evaluation of serum samples from our previous animal experiment suggested these is no existence of pGM-CSF in serum sample obtained from PRRSV-JXA1-infected piglets whereas upregulated pGM-CSF was only observed in sera of PRRSV-JXA1-infected piglets after the received therapeutic mAb treatment against PRRSV infection and increased pGM-CSF levels in serum aligned with reduced pneumonia incidence in mAb-treated animals. These results implying that pGM-CSF might play a host-protective role against PRRSV infection *in vivo* rather than *involve in* HP-PRRSV induced cytokine storms, which appears to be similar to GM-CSF’s effects during influenza A virus infection (39).

Besides above observations, previous research demonstrated that piglets vaccinated with a recombinant PRRSV-MLV bearing pGM-CSF exhibited lower viremia, fewer gross lesions in lungs, and higher levels of IFN-γ secretion after challenging with HP-PRRSV (22), suggesting that pGM-CSF may enhanced Th1 mediated response during PRRSV-MLV immunization and sequential HP-PRRSV challenged (22). In this study, upregulated serum pGM-CSF level and IFN-γ, along with M1-biased genes expression in PAMs were observed in PRRSV-JXA1-infected piglets after the received therapeutic mAb treatment to block HP-PRRSV inoculation. Therefore, these data further suggested that pGM-CSF might play a positive role to activate immune system against PRRSV infection and consisted with Th1 type immune response. Moreover, one of our previous research demonstrated that immunization of mice with PRRSV-specific IgM adjuvanted inactivated PRRSV vaccine (KIV) enhanced cell mediated immunity (CMI) as evidenced by significant increasing of IFN-γ producing cells from splenocytes obtained from immunized mice (35). Consistently, our latest *in vivo* experiment in piglets demonstrated that piglets immunized with this novel PRRSV-specific IgM adjuvanted PRRSV-KIV produced higher level of IFN-γ (54) in serum, further implying a link between serum pGM-CSF level and Th1 type response alone with potential M1-like activation in macrophage.

The *in vivo* source of serum pGM-CSF in therapeutic mAb-treated piglets remains unclear, since upregulation of pGM-CSF mRNA expression in PAMs isolated from piglets demonstrated no significant differences among control, PRRSV-JXA1-infected, and therapeutic mAb-treated groups based on transcriptome data and qPCR (**Figure 7**). As a possible explanation, early differences in GM-CSF mRNA expression among experimental groups may have disappeared at later time points (21 dpi), when PAMs were collected from piglets; by then, transcriptional levels of pGM-CSF in PAMs had returned to normal and only slight differences were observed among groups. Alternatively, PBMCs may be another *in vivo* source for pGM-CSF production in piglets which may mirror the scenario of COVID-19 patients as increased percentages of GM-CSF-producing leukocytes have been found in blood of COVID-19 patients (43). Therefore, it is possible that PAMs may act as *in vivo* effector cells rather than producing cells of pGM-CSF. Meanwhile, GM-CSF is proposed to exert a pro-inflammatory effect as GM-CSF-treated monocytes/macrophages differentiate into “M1-like” polarized cells with characteristics of both M1 and M2 cells (41). Therefore, PAMs transcriptome data for pGM-CSF-activated genes (both M1-like and M2-like genes) were mined from previously obtained transcriptome data and suggested that expression of certain genes downstream of GM-CSF classified as M1-like cytokines (41) were preferentially upregulated and activated in PAMs collected from piglets receiving therapeutic mAb, aligning with increased serum pGM-CSF levels in mAb-treated animals. Thus, pGM-CSF-induced activation of PAMs caused them to assume an M1-like state that may have reduced PRRSV pathogenesis.

Inhibition of IFNs-mediated innate immunity by PRRSV infection is considered a major factor contributing to PRRSV pathogenesis and PRRSV genome encodes several IFNs antagonists that block IFN induction and IFN-activated JAK/STAT signaling (55–58). However, piglets inoculated with the PRRSV-A2MC2-p90 strain, an attenuated PRRSV vaccine candidate with an IFNs-inducing phenotype (59, 60), produced high titers of serum PRRSV-specific neutralizing antibodies (NAbs) and were protected against heterogeneous PRRSV challenge (61). Since both IFNs and pGM-CSF were inhibited at post-transcription level, removing of PRRSV mediated antagonism for GM-CSF mRNA translation to generate bio-active pGM-CSF may offer alternative way to enhance MLV vaccine efficiency.

In conclusion, here a pGM-CSF ELISA with high sensitivity, specificity, and repeatability was developed. Use of this ELISA assay uncovered a discrepancy between pGM-CSF mRNA and protein levels in PRRSV-infected PAMs as evidence that PRRSV infection mechanistically exerted post-transcriptional inhibition of pGM-CSF production. Nevertheless, results of evaluation of pGM-CSF protein levels in piglet sera and activation of downstream signaling of pGM-CSF in PAMs were consistent with observed reduced levels of intracellular PRRSV-RNA and decreased pneumonia incidence in therapeutic mAb-treated piglets, suggesting that pGM-CSF and its associated effects may protect piglets from PRRSV pathogenic processes. Thus, pGM-CSF is a novel target for improving PRRSV MLV immunization or incorporated as an adjuvant in anti-PRRSV vaccines.

## Data availability statement

The datasets presented in this study can be found in online repositories. The names of the repository/repositories and accession number(s) can be found in the article/**Supplementary Material**.

## Ethics statement

The animal study was reviewed and approved by Animal Welfare Committee of Northwest A&F University.

## Author contributions

CW designed this study. QJ, GQ, BL, YB, GW, RC, XZ, ZZ, and YY conduct the experiments. YY and CW analyze the data. CW secure the funds. YY and CW prepared the main body of this manuscript. CW revised the manuscript. All authors contributed to the article and approved the submitted version.

## Funding

This work was supported by a grant from the National Natural Science Foundation of China awarded to CW (No. 32172842) and a grant from the Natural and Science Development Program of Shaanxi Province awarded to CW (Grant No. 2020JM-158).

## Conflict of interest

Author RC was employed by the company Shaanxi Innolever Biotechnology Co., Ltd.

The remaining authors declare that the research was conducted in the absence of any commercial or financial relationships that could be construed as a potential conflict of interest.

## Publisher’s note

All claims expressed in this article are solely those of the authors and do not necessarily represent those of their affiliated organizations, or those of the publisher, the editors and the reviewers. Any product that may be evaluated in this article, or claim that may be made by its manufacturer, is not guaranteed or endorsed by the publisher.

## Supplementary material

Supplementary Figure 1 Expression of recombinant pGM-CSF and screening anti-pGM-CSF monoclonal antibody. **(A)** Electrophoresis of pGM-CSF cDNA cloned from total cDNA reverse transcribed from total RNA isolated from LPS-stimulated PBMCs of piglet. **(B)** Analysis of purity for recombinant pGM-CSF expressed in *E. coli*. using SDS-PAGE.

Supplementary Figure 2 Characterization of mAb-2A4H11 and rabbit polyclonal antibody against pGM-CSF for pGM-CSF expressed in mammalian cells. **(A)** BHK21 cells were transfected with pVenus-pGM-CSF plasmid for 24 hours. Then the cells were fixed and permeabilized before antibodies staining using mAb-2A4H11 and an isotype control mAb. Interaction between mAb with corresponding target was visualized by secondary antibodies. **(B)** BHK21 cells were transfected with pVenus-pGM-CSF or empty vector for 24 hours. Then the cells were harvested for SDS-PAGE followed by western blotting assay use mAb-2A4H11, GFP-specific-mAb and purified pGM-CSF rabbit polyclonal antibody to confirm the reactivity.
