# Supplementary figures and images for: Evaluation of porcine GM-CSF during PRRSV infection in vitro and in vivo indicating a protective role of GM-CSF related with M1 biased activation in alveolar macrophage during PRRSV infection
Source: Front Immunol. 2022 Oct 19;13:967338. doi: 10.3389/fimmu.2022.967338 (PMC9627285; doi:10.3389/fimmu.2022.967338)

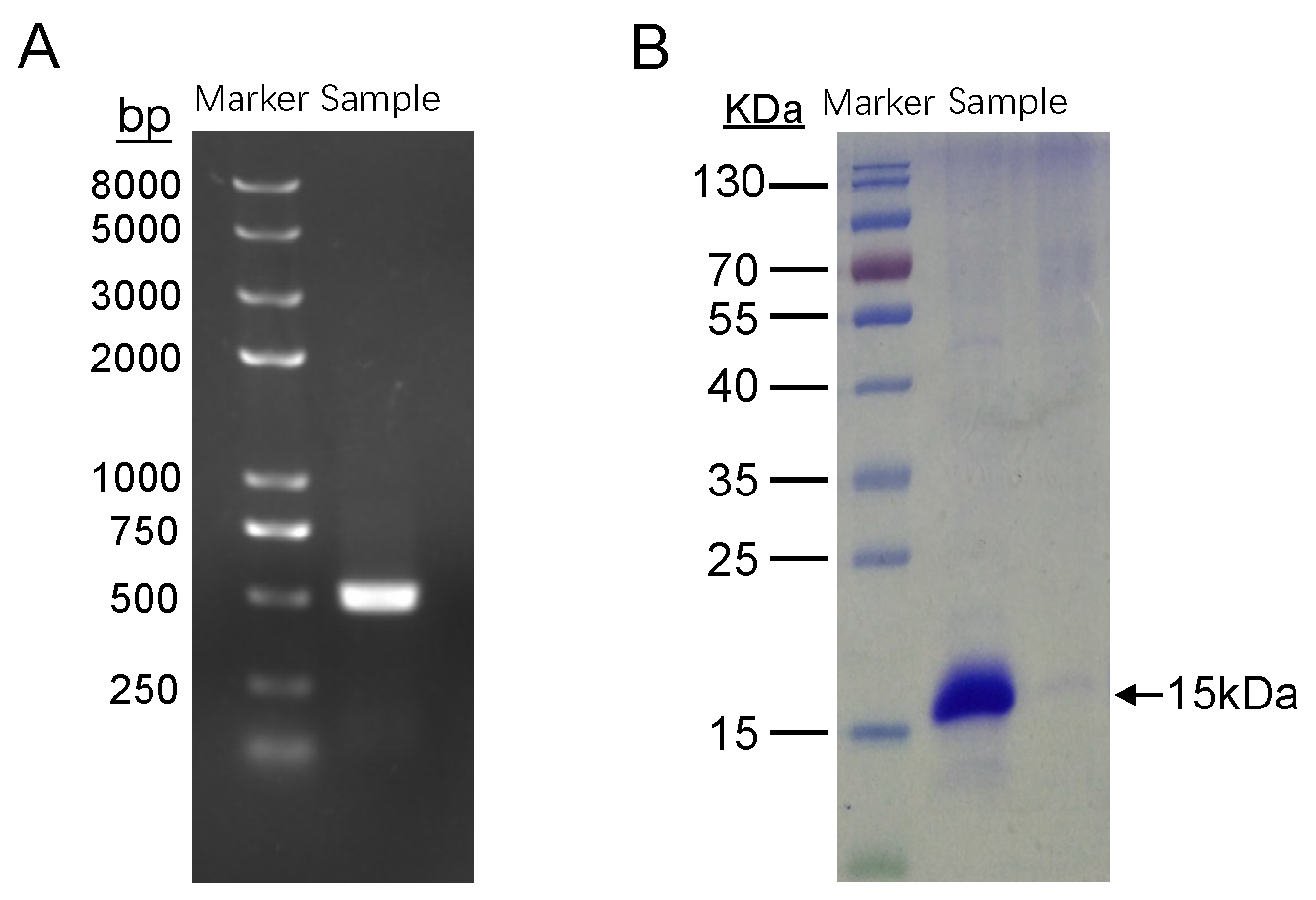

Supplement: Supplementary Figure 1 — Expression of recombinant pGM-CSF and screening anti-pGM-CSF monoclonal antibody. (A) Electrophoresis of pGM-CSF cDNA cloned from total cDNA reverse transcribed from total RNA isolated from LPS-stimulated PBMCs of piglet. (B) Analysis of purity for recombinant pGM-CSF expressed in E. coli. using SDS-PAGE. [file Image_1.tiff]

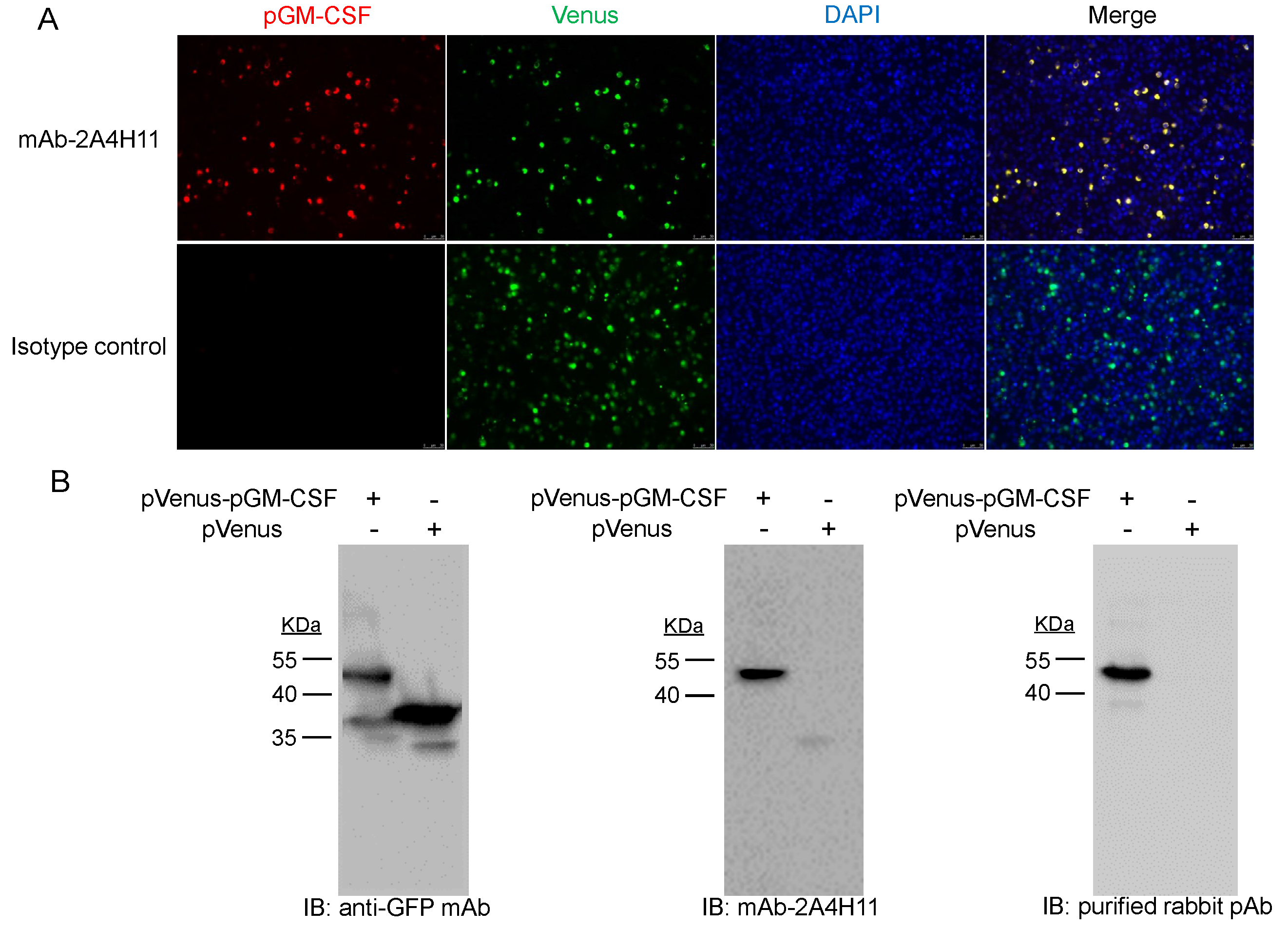

Supplement: Supplementary Figure 2 — Characterization of mAb-2A4H11 and rabbit polyclonal antibody against pGM-CSF for pGM-CSF expressed in mammalian cells. (A) BHK21 cells were transfected with pVenus-pGM-CSF plasmid for 24 hours. Then the cells were fixed and permeabilized before antibodies staining using mAb-2A4H11 and an isotype control mAb. Interaction between mAb with corresponding target was visualized by secondary antibodies. (B) BHK21 cells were transfected with pVenus-pGM-CSF or empty vector for 24 hours. Then the cells were harvested for SDS-PAGE followed by western blotting assay use mAb-2A4H11, GFP-specific-mAb and purified pGM-CSF rabbit polyclonal antibody to confirm the reactivity. [file Image_2.tiff]
