# Supplementary material for: Evaluation of porcine GM-CSF during PRRSV infection in vitro and in vivo indicating a protective role of GM-CSF related with M1 biased activation in alveolar macrophage during PRRSV infection
Source: Front Immunol. 2022 Oct 19;13:967338. doi: 10.3389/fimmu.2022.967338 (PMC9627285; doi:10.3389/fimmu.2022.967338)
Supplement: Supplementary file 3 [file DataSheet_1.docx]

**Supplementary Table 1.** List of siRNA and corresponding sequence used in this study

| siRNA | Sequence (5'-3') |  |
| --- | --- | --- |
| siRNA-1 sense | CCCAGUCUAUCACCUUCAATT |  |
| siRNA-1 antisense | UUGAAGGUGAUAGACUGGGTT |  |
| siRNA-2 sense | GACGUCGUCUGUGAAAUGUTT |  |
| siRNA-2 antisense | ACAUUUCACAGACGACGUCTT |  |
| siRNA-3 sense | UACUGUUGUUUAGAAGGCUCAGGGCUU |  |
| siRNA-3 antisense | AAGCCCUGAGCCUUCUAAACAACAGUA |  |
|  |  |  |
