# Supplementary material for: Evaluation of porcine GM-CSF during PRRSV infection in vitro and in vivo indicating a protective role of GM-CSF related with M1 biased activation in alveolar macrophage during PRRSV infection
Source: Front Immunol. 2022 Oct 19;13:967338. doi: 10.3389/fimmu.2022.967338 (PMC9627285; doi:10.3389/fimmu.2022.967338)
Supplement: Supplementary file 4 [file DataSheet_2.docx]

**Table 2.** List of primers and corresponding sequence used in this study

| Primers | Sequence (5'-3') | Description |
| --- | --- | --- |
| pGM-CSF-F1 | CGGAATTCATGTGGCTGCAGAACCTG | Cloning of pGM-CSF |
| pGM-CSF-R1 | CCGCGGCCGCTTACTTTTTGACTGGCCCCCA |  |
| pGM-CSF-F2 | ATGCCATCAAAGAAGCCCTGA | qPCR for pGM-CSF mRNA |
| pGM-CSF-R2 | GCTTGTACAGGTTCAGGCGA |  |
| GAPDH-F | CCTTCCGTGTCCCTACTGCCAAC | qPCR for GAPDH mRNA |
| GAPDH-R | GACGCCTGCTTCACCACCTTCT |  |
| iNOS-F | GCACCTGCGTTATGCCACCAAC | qPCR for iNOS mRNA |
| iNOS-R | TGAGCTGAGCGTTCCAGACCC |  |
| IFN-γ-F | TCACTGATGGCTTTGCGCTG | qPCR for IFN-γ mRNA |
| IFN-γ-R | AGAGCATGATCCGAGACGTG |  |
| TNF-α-F | AGAGCATGATCCGAGACGTG | qPCR for TNF-α mRNA |
| TNF-α-F | CAGTAGGCAGAAGAGCGTGG |  |
| IL-6-F | ACAAAGCCACCACCCCTAAC | qPCR for IL-6 mRNA |
| IL-6-R | CGTGGACGGCATCAATCTCA |  |
| CD163-F | TCCTTGTGGGATTGTCCTGC | qPCR for CD163 mRNA |
| CD163-F | AGGGATTCTCGGCTCTTTGC |  |
| IL-4-F | CTTCGGCACATCTACAGACACC | qPCR for IL-4 mRNA |
| IL-4-R | CTTCATAATCGTCTTTAGCCTTTCC |  |
| IL-13-F | GGTCAATATCACCCAGAACCAGAAG | qPCR for IL-13 mRNA |
| IL-13-R | TGCAGTCGGAGATGTTGATGAGG |  |
| IL-12-F | TACCACTTGAACTAGCCACGAAT | qPCR for IL-12 mRNA |
| IL-12-R | CTAAGGCACAGGGTTGTCATAAA |  |
| CCL17-F | ATGCAGCTCGAGGAACCAAC | qPCR for CCL17 mRNA |
| CCL17-R | GTCACAAGCACAATGGCGTC |  |
| IL-1β-F | GACCCCAAAAGATACCCAAA | qPCR for IL-1β mRNA |
| IL-1β-R | TCTGCTTGAGAGGTGCTGATG |  |
| TGF-β-F | TCCAAGGACCCTTCTCGGAT | qPCR for TGF-β mRNA |
| TGF-β-R | AAAAACCGAGATGGGCGAGA |  |
| MGL-1-F | ACTTCTCCGGCATGGTTCTG | qPCR for MGL-1 mRNA |
| MGL-1-R | GTTGAGCACTTTCGCAGCAA |  |
| IRF-4-F | CCGTCATTAGTGCGTCAGTTCT | qPCR for IRF-4 mRNA |
| IRF-4-R | TTGCAGCCCACAAAAAGCA |  |
| IL-10-F | CGGCGCTGTCATCAATTTCTG | qPCR for IL-10 mRNA |
| IL-10-R | CCCCTCTCTTGGAGCTTGCTA |  |
| PRRSV-F | CGGCAATTGTGTCTGTCGTC | Amplification of PRRSV-N protein based Replicon for probe |
| PRRSV-R | GGCAAACTAAACTCCACAGTG |  |
| PRRSV-probe | CTCCCTGAATCTGACAGGG | For Taqman probe |
|  |  |  |
